# Effect of Two Transport Options on the Welfare of Two Genetic Lines of Organic Free Range Pullets in Switzerland

**DOI:** 10.3390/ani8100183

**Published:** 2018-10-19

**Authors:** Helena Sprafke, Rupert Palme, Paul Schmidt, Michael Erhard, Shana Bergmann

**Affiliations:** 1 Department of Veterinary Sciences, Chair of Animal Welfare, Ethology, Animal Hygiene and Animal Husbandry, Faculty of Veterinary Medicine, LMU Munich, Veterinärstraße 13/R, DE-80539 Munich, Germany; m.erhard@tierhyg.vetmed.uni-muenchen.de (M.E.); s.bergmann@lmu.de (S.B.); 2 Department of Biomedical Sciences, University of Veterinary Medicine, Veterinärplatz 1, A-1210 Vienna, Austria; Rupert.Palme@vetmeduni.ac.at; 3 Statistical Consulting for Science and Research, Zimmerstr. 10, DE-76327 Pfinztal, Germany; paul@statistische-modellierung.de

**Keywords:** animal welfare, transport, pullet, stress parameter, corticosterone metabolite

## Abstract

**Simple Summary:**

Animal welfare has been of increasing interest to consumers and producers of animal products in Europe. Issues during transport affect both the wellbeing and the productivity of livestock. This study was conducted to analyze two practice-oriented transport variants of organically mixed-held white and brown pullets. No significant difference could be found between the transport variants. Instead, we discovered clear differences between the two genetic pullet lines.

**Abstract:**

The welfare of two genetic lines of organic layer hen pullets—H&N Super Nick (HNS) and H&N Brown Nick (HNB)—was compared during two commercial transport variants of 15 flocks of mixed-reared birds. Birds were either transported overnight (with a break in travel), or were transported direct to the layer farm (without a break in travel). Samples of feces were collected non-invasively from 25 birds of each genetic line per flock for each transport variant before transportation to evaluate baseline values of glucocorticoid metabolites, and at 0 h, 3 h, 6 h, 10 h, 24 h, 34 h, 48 h, 58 h, and 72 h after the end of transportation, to measure transportation and translocation stress. We assessed the fear toward humans with the touch test before transportation, and we checked the birds’ body condition by scoring the plumage condition and the occurrence of injuries. Body weight before and weight loss after transportation were determined, and ambient temperature was measured before, during, and after transportation. Stress investigations showed no significant differences between the transport variants (effect: −0.208; 95% confidence interval (CI): (−0.567; 0.163)). Instead, we discovered differences between the pullet lines (effect: −0.286; 95% CI: (−0.334; 0.238)). Weight loss was different between the transport variants (2.1 percentage points; 95% CI: (−2.6; −1.5)) and between the genetic lines, as HNB lost significantly less weight than HNS (0.5 percentage points; 95% CI: (0.3; 0.7)).

## 1. Introduction

The World Organization for Animal Health provides a reference document [1] of international standards for animal health and zoonosis; it grants animals kept under human care the internationally recognized "five freedoms" of welfare described as follows: freedom from hunger, thirst and malnutrition; freedom from fear and distress; freedom from physical and thermal discomfort; freedom from pain, injury and disease; freedom to express normal behavior patterns.

The Swiss consumer assumes that organic livestock farming applies more species-appropriate animal husbandry corresponding to the animals’ natural living conditions, and thus provides better animal welfare than conventional farming practices [2]. Animal transport is an exceptional situation, because animal welfare may be compromised for a certain time. The European Commission Regulation (EC) No 1/2005 [3], limits the transport duration of poultry to less than 12 h. The Swiss Animal Welfare Regulation has limited the maximum duration of animal transport to less than 8 h since its amendment to Art. 152a (1) of 28 October 2015 [4], like the German Animal Welfare Transport Regulation [5]. Whereas some of the German organic labels further limit the maximum transport durations to less than 4 h or 2 h [6,7,8], none provide specific requirements for poultry, pullets, or laying hens. Similarly, the Swiss organic labels Bio Suisse [9] and KAG (Consumer-Working-Group; German designation: Konsumenten-Arbeits-Gruppe) [10] refer to the transportation guidelines of the Swiss Animal Protection [11], which are designed for big and small animals, not including economic fowl. All regulations, including EC No 1/2005, disregard loading and unloading time (Table 1).

Various stressors such as climate, environment, nutrition, physical, and social and physiological conditions are likely to influence welfare and performance [12]. The present study examined the effects of transportation and translocation on the stress hormone levels of 18-week-old organically reared pullets of two mixed-held layer lines—H&N Super Nick and H&N Brown Nick—in transit from the rearing farm to the farm of laying hens (novel environment) in Switzerland. The geographically dispersed locations of rearing and laying farms require that hens are transported on the road over distances of varying lengths. We compared two practice-oriented transport variants. Although the transportation process is supposed to be the most stressful environmental challenge experienced by broilers [13], catching and handling of the birds may already have profound effects on the degree of physiological stress, and it may reduce welfare during the upcoming transit [14,15,16]. Thus, we took into account the entire duration of the hens being held in plastic crates, including loading and unloading time. 

To evaluate the stress response to the two transport variants, we measured corticosterone metabolites non-invasively in excretions of the pullets. Fecal corticosterone metabolites have previously been used as a reliable indicator of adrenocortical activity [17].

The project was initiated by Switzerland’s largest organic egg supplier—hosberg AG in Rüti—and was supported by two other Swiss-based companies—Wüthrich Brüterei AG in Belp and Prodavi AG in Schötz. Participation in “LTK (Institute of Laboratory Animal Sciences) Module 2: Training for Persons Responsible for Directing Animal Experiments” was required as a prerequisite for the intercantonal approval by the Swiss Cantonal Veterinary Office in Zurich, and the project was approved on 6 January 2016, under registration number ZH196/15. The study refers to Europe, with special emphasis on Switzerland.

## 2. Animals, Materials and Methods

### 2.1. Experimental Design

The study was conducted with organically mixed-held pullets of two genetic lines—H&N Super Nick (HNS) and H&N Brown Nick (HNB)—of a commercial breeder and distributor (H&N International, Cuxhaven, Germany). Parent animals were imported to Switzerland and raised as organic laying hens. The experimental unit consisted of pullets and laying hens of 15 flocks, which were reared and kept according to the guidelines of Bio Suisse (Association of Swiss Organic Farming Organizations, Basel, Switzerland) [9] on free-range farms. Each rearing farm raised 4000 pullets, and farms of laying hens kept 2000 birds. The average ratio between HNS and HNB normally was 50:50 to 60:40. The transport to the farm of laying hens was realized at the age of 18 weeks. The study was based on two practically relevant commercial transport variants—with and without transportation break—which were categorized according to distance and length. Variant I “transport overnight” (transportation was performed with break) was compared with Variant II “direct transport” (transportation was performed without break). On average, 2014 birds were transferred on each transit. Each plastic crate (90.5 × 61.5 × 31.5 cm) was loaded with 16 pullets according to the Swiss Order on the Protection of Animals [4]. Because the start of loading also means the starting point of stress, we defined the time from the beginning of loading until the end of unloading as “time in plastic crate” or transport duration. Thus, the average transport duration was 13.5 h for Variant I and 5.0 h for Variant II, whereas the mean journey time alone was 2.6 h for Variant I and 1.0 h for Variant II (time on the road). Loading regularly began at 7 p.m. The legally prescribed transport duration was never exceeded [4]. We timed our investigation to include winter, spring, and summer.

Temperature was measured with HOBO U10 (temperature data loggers, Onset Computer Corporation, Bourne, MA, USA) inside of the stable at animal head height and during transportation inside of the plastic crates on the upper edge. For both transport variants, temperature was recorded during the whole investigation period every 10 min per flock. Means of minimum and maximum temperature values during the testing period (January until July) for Variant I ranged from 11.1 to 32.3 °C (rearing farm), 1.9 to 34.7 °C (transportation vehicle) and 3.8 to 34.1 °C (farm of laying hens) and for Variant II from 7.6 to 26.3 °C (rearing farm), −8.9 to 28.8 °C (transportation vehicle), and 6.1 to 26.2 °C (farm of laying hens). 

Animal husbandry varied according to the individual farmer’s management.

### 2.2. Corticosterone Monitoring

To examine the effects of transportation and translocation on stress in each flock, corticosterone levels were measured non-invasively by extracting metabolites from bird droppings. For each sampling time point, 25 pullets of each genetic line were randomly caught from different tiers of the dimmed barn. To enable the collection of individual, spontaneously voided droppings, the pullets were placed separately in cleaned and disinfected plastic crates, and marked on their legs with a pen (Edding-Egg-Color-Pen, Wunstorf, Germany). Samples were collected within 1 h of the experimenter entering the barn according to Rettenbacher et al. [21], who found a first major peak 1 h after a stress pulse in laying hens. One dropping per bird was collected immediately after defecation, put into frost-resistant plastic bags and frozen on dry ice at a usual temperature of −78.5 °C. Droppings were transferred to a freezer after sampling. To determine baseline concentrations, pullets were sampled at 9:00 a.m., two days before transportation. For measurements of transportation and translocation stress, further droppings in both variants were collected 0 h, 3 h, 6 h, 10 h, 24 h, 48 h and 72 h after transportation. Initially, the flocks had been sampled 9 h and 12 h (instead of 10 h) after transportation. However, at these time points, only a few (if any) birds defecated. To prevent an unworkable additional work load and a possible violation of the numerical limit of permitted experimental birds, we decided to collect samples 10 h after transportation. Taking the circadian rhythm into account, flocks of Variant II were sampled additionally 34 h and 58 h after transportation. Altogether, 5751 droppings were collected and analyzed.

In the laboratory, 0.5 g of each sample was suspended in 5 mL of 60% (*v*/*v*) methanol by shaking for 30 min on a multi-vortex (RapidVap, Labconco, Kansas City, MO, USA) [22]. When a smaller portion had to be used, an aliquot of methanol was added. After centrifugation (GS-6KR Centrifuge, Beckman, Krefeld, Germany) for 15 min, aliquots of the supernatant were diluted 1:10 with assay buffer, and concentrations of fecal corticosterone metabolites (CM) were determined with a cortisone enzyme immunoassay [21]. The applied method has been validated physiologically and biologically for chickens by Rettenbacher et al. [21,23].

### 2.3. Hen-Human Relationship: Touch Test

The level of fear of humans is an important determinant of welfare of pullets and laying hens. Regular handling of pullets is fear reducing [24], and positive additional human contact of laying hens reduces their fear level and influences their corticosterone level in blood positively [25]. In contrast, fear-inducing humans reduce the wellbeing of animals [26]. Accordingly, we tested each flock on avoidance and approach behavior by using the touch test of Raubek et al. [27] to assess the birds’ reaction to an unfamiliar human. The test was performed with each flock by the same test person, who was unfamiliar to the flock before test. The test person wore protective clothing such as a blue overall, plastic overshoes and a hair cloth. Tests were carried out in the roofed outdoor run area (winter garden) of the rearing farm. Entering the winter garden was the initial contact between flock and test person. The unfamiliar test person moved slowly—one step per second—through the winter garden, approached a group of at least three pullets, squatted for 10 s and then counted all pullets within one arm’s length around her. Thereafter, the test person tried to touch one bird after the other. The test was carried out until 33 groups were examined. Any attempt to approach a group or squat down was counted, even if all pullets retreated from the test person [28].

### 2.4. Body Condition

The body condition of the birds was evaluated by scoring the plumage and integuments before and after transportation, following feces sampling and the touch test. The assessment basis was the LayWel grading scheme [29] modified according to Schwarzer et al. [30] for pullets. Plumage condition was divided into four degrees of severity (4 = no damage, 3 = 1–5 damaged feathers, 2 = >5 damaged feathers, 1 = plucked area >1 cm). A higher score equaled a better plumage condition. This was assessed on seven individually scored body areas, resulting in a maximum pooled score of 28. Damage of flight feathers, tail feathers, and the presence of fault bars were evaluated separately with binary scores (0 = negative and 1 = positive). Originating from a total plumage score of 28, a bad feather cover was indicated by ≤11–14, and a good feather condition by ≥18–20. Injuries were divided into three degrees of severity (0 = negative, 1 = Ø ≤0.5 cm, 2 = Ø >0.5 cm) on 10 individually scored body areas. Injuries of the comb, head and eyelid were evaluated separately with binary scores (0 = negative and 1 = positive).

### 2.5. Live Weight

To check minimum body weight, which is 1300 g for HNS and 1479 g for HNB at the age of 18 weeks (according to the breeder and distributor H&N International), 50 numerically marked birds, 25 per line, were weighed during loading. To check a possible transport-related weight reduction due to water and feed withdrawal and “time in plastic crates,” we compared body weights before and after transportation (following sampling) for each hen. Results were compared with a control flock that was not transported and was kept overnight in the winter garden without access to water and feed but were free to move around; hens of the control flock were weighed in the evening (8:00 p.m.) and in the morning (7:00 a.m.). For evaluation, the weight of the same numerically marked bird was compared in each case. Weight determination was carried out with a BAT1 poultry scale (VEIT Electronics, Moravany, Czech Republic). The 50 hens of the transport study were divided into four transport crates. To ensure a regular number of 16 birds per crate according to the Swiss Order on the Protection of Animals [4], the transport crates were supplemented with non-weighed birds.

### 2.6. Statistical Analysis

For the statistical analysis, the relationships between the predictors (transport variant, layer line, flock) and the response variables (baseline CM concentration, CM concentration after transport, returned to baseline value after 72 h, and difference in plumage score before and after transport, transport weight) were analyzed simultaneously using mixed-effects models. The flock was modeled as an unstructured random effect for the model constant (intercept), and the transport variant and the layer line were modeled as ordinary fixed effects. For the continuous response baseline CM concentration, CM concentration after transport, difference in plumage score before and after transport, and transport weight, normal distributions were chosen as observation models. For the binary outcome return to baseline value after 72 h, a logistic regression model was used. Results from this analysis were expressed as odds ratios (OR). For baseline CM concentration, temporal progression was also considered by including time as an unstructured random effect (in contrast to a temporal effect, because of very few unequally distributed time points). 

Data were analyzed by using the statistical programming language R [31]. All (generalized) mixed-effects models were estimated by the integrated nested Laplace approximation approach [32] within a fully Bayesian setup.

## 3. Results

### 3.1. Corticosterone Monitoring

Mean baseline concentrations of excreted CM in the examined flocks were 43 ng/g and 66 ng/g for Variant I and Variant II, respectively. Overall, birds of Variant II had higher baseline values than birds of Variant I (Table 2). However, differences were not significant (effect: −18.6; 95% confidence interval (CI): (−45.3; 9.1)).

**Figure 1:**
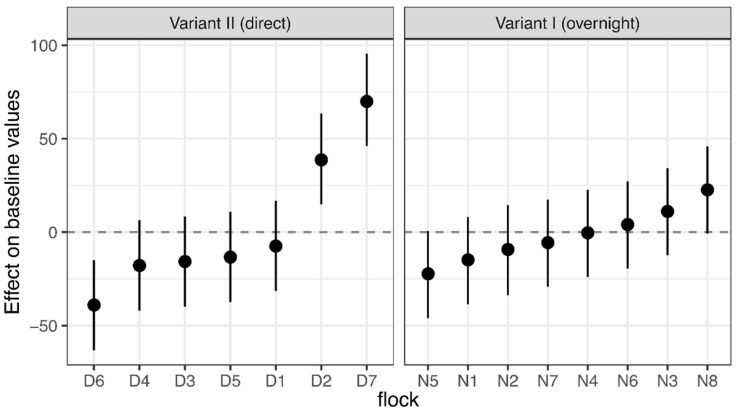
Linear mixed-effects model. Effect of the flock on the baseline concentrations of corticosterone metabolites in either two transport variants, modeled with the flock as an unstructured random effect for the model constant, and with the transport variant and the layer line as fixed effects. The figure shows estimated values (circles) with their 95% confidence intervals (bars) of the effect of flock on baseline concentrations. Estimates that do not cross zero deviate significantly from the baseline concentration.

HNB individuals had significantly lower baseline values than HNS birds (effect: −37.8; 95% CI: (−45.4; −30.3)). Three flocks showed significant deviations from mean baseline values in Variant II: two flocks with significant above-average values (D2 and D7) and one with significantly lower values (D6). Any effect that crossed zero did not significantly deviate from the mean baseline value in this flock (Figure 1).

The transit from the rearing farm to the farm of the laying hens resulted in higher mean concentrations of CM compared with the mean baseline values. The highest values were found immediately on arrival (0 h). The mean concentration at 0 h was 173 ng/g and 323 ng/g for Variant I and Variant II, respectively. CM concentrations decreased rapidly during the 0–6 h interval after transportation. Variant I showed an increase during the 6–12 h interval, followed by a steady decline. Values for Variant II were slightly increased at 24 h, 48 h, and 72 h, and slightly decreased at 34 h and 58 h, with the additional sampling times considering the circadian rhythm (Table 3).

**Figure 2:**
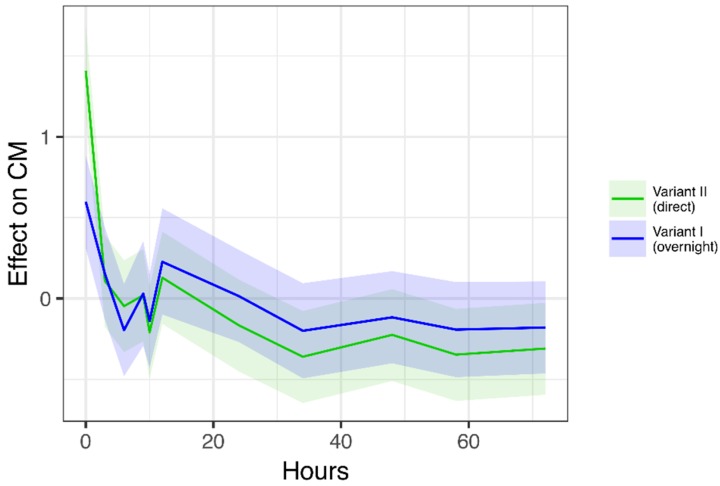
Linear mixed-effects model. Effect of time after transportation (hours) on concentrations of corticosterone metabolites (CM), considering the flock as a random effect, and the transport variant and the layer line as fixed effects. Estimated temporal progression of CM concentrations, shown as estimated effects (solid lines) and 95% confidence intervals (shaded areas) for each transport variant.

We found no significant difference in CM concentrations between Variant I and Variant II, because the overall trend was similar, and confidence intervals overlapped strongly (effect: −0.208; 95% CI: (−0.567; 0.163)) (Figure 2).

**Figure 3:**
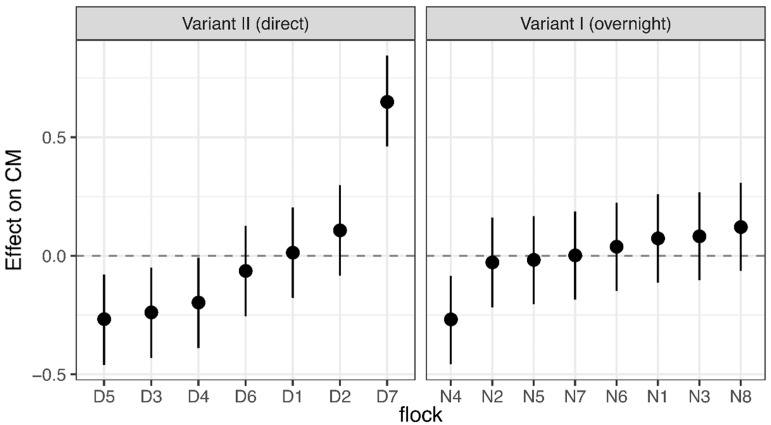
Linear mixed-effects model. Effect of the flock on concentrations of corticosterone metabolites (CM) 0–72 h after transportation in either two transport variants, modeled with the flock as an unstructured random effect for the model constant, and with the transport variant and the layer line as fixed effects. The figure shows estimated values (circles) with their 95% confidence intervals (bars) of the effect of flock on CM concentrations. Estimates that do not cross zero deviate significantly from the mean value.

Significant differences in CM concentrations were found once again between the layer lines. HNS had higher values than HNB (effect: −0.286; 95% CI: (−0.334; −0.238)). Furthermore, considerable variations among flocks were found. Significant above-average values could be measured for one flock of Variant II (D7), significantly lower values for three flocks of Variant II (D5, D3 and D4), and one flock of Variant I (N4) during the 0–72 h interval. Any effect that crosses zero does not significantly deviate from the mean CM concentration in this flock (Figure 3).

**Figure 4:**
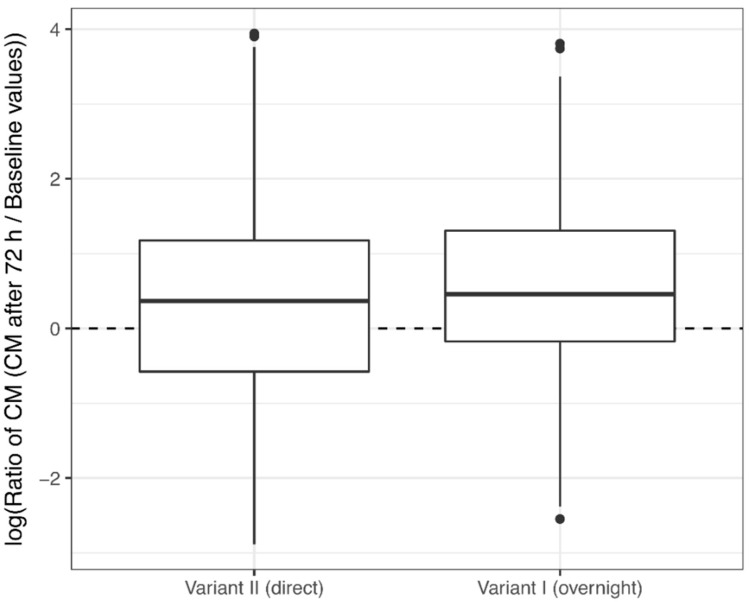
Ratio of concentrations of corticosterone metabolites (CM) after 72 h to CM baseline concentrations in two transport variants. Thick black lines show mean ratios, boxes represent upper and lower quartiles, whiskers represent 95% confidence intervals, and dots show outliers. A mean ratio above zero indicates an increase in CM concentration relative to the baseline value, whereas a mean ratio below zero indicates a decrease.

The ratio of CM concentrations after 72 h to CM baseline values showed that most CM concentrations did not return to baseline values in both transport variants (Figure 4). For both layer lines, we could not find a significant effect of transport variant on the return to baseline values (effect: 0.43; 95% CI: (−2.41; 3.26) and −1.49; 95% CI: (−4.34; 1.37) for HNS and HNB, respectively).

### 3.2. Hen-Human Relationship: Touch Test

With the touch test, we evaluated the hen–human relationship based on the approach and avoidance behavior of the birds between flocks. To evaluate whether this behavior was reflected in the measured CM concentrations, we compared CM concentrations between hens that stayed an arm length away from the examiner, and those that could be touched. An increase in CM concentration by one unit (1.0 ng/g) resulted in a significantly greater number of hens that could be touched (effect: 0.004; 95% CI: (0.001; 0.006)). In addition, a few differences in approach and avoidance behavior between flocks were found: One flock (D5) had significantly fewer hens that could be touched compared with three other flocks (D2, D6, and N2).

### 3.3. Body Condition

**Figure 5:**
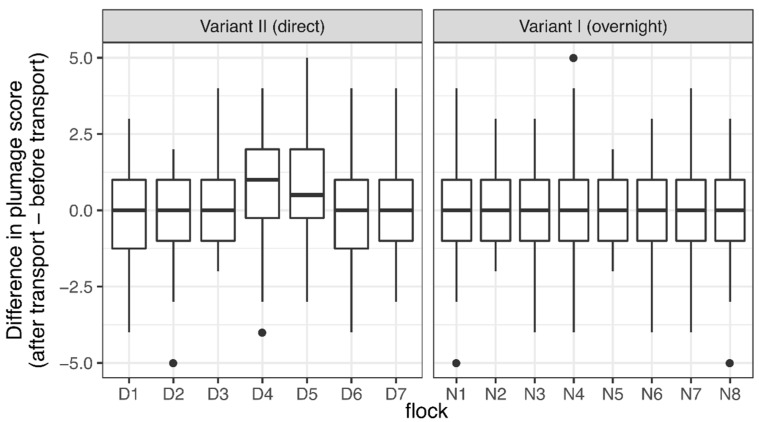
Difference across flocks in plumage score before and after transportation in two transport variants. Thick black lines show mean values, boxes represent upper and lower quartiles, whiskers represent 95% confidence intervals, and dots show outliners. A mean value above zero indicates a better plumage score after than before transportation.

The examined flocks of both transport variants showed an average plumage score of 24.62 ± 1.37 (mean ± SD) before and after transportation, indicating a good feather condition (maximum possible score = 28, for seven body areas with four degrees of severity). Flocks D4 and D5 of Variant II had a better plumage score after transportation than before (Figure 5).

Altogether, we found no significant differences in the plumage condition of the body areas scored with four degrees of severity before and after transportation, regardless of layer line, transport variant or flock, with one exception: HNB in comparison with HNS showed less plumage deterioration (−0.28, 95% CI: (−0.8; 0.25)) in Variant I, and in Variant II, greater deterioration (0.35, 95% CI: (−0.03; 0.73)). The total plumage score, including those body areas scored with two forms of severity (flight and tail feathers, fault bars) apparently significantly improved after transportation compared with before transportation in Variant II (OR: 0.672; 95% CI: (0.53; 0.863)) but not in Variant I (OR: 1.454; 95% CI: (0.931; 2.218)). Integument injuries of body areas scored with three degrees of severity were not sufficiently variable in their distribution of characteristics, and only isolated injuries were found. The same applies for integument injuries of the eyelid (binary score). Both comb and head (evaluated with a binary score) were scored positive in 13% and 4% of the cases, respectively. We could find no major differences in integument injuries before and after transportation.

**Figure 6:**
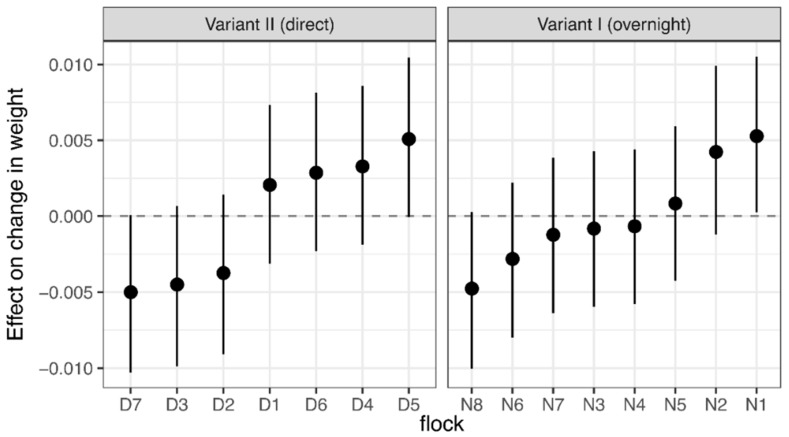
Linear assorted regression model. Estimated body weight differences across flocks before and after transportation in two transport variants, considering the factors of the layer line, the transport variant, and the average temperature on the rearing farms. The figure shows estimates (solid circles) and 95% confidence intervals (bars) of the effect of flocks on body weight changes. Estimates that cross zero do not deviate significantly from the mean values.

Following the transit from the rearing farm to the farm of laying hens, the birds showed a weight loss of −2.9% ± 1.9% (mean ± SD). Comparing both transport variants, birds of Variant I lost significantly more weight (2.1 percentage points; 95% CI: (−2.6; −1.5)) than birds of Variant II. Regarding the layer lines, HNB lost significantly less weight than HNS (0.5 percentage points; 95% CI: (0.3; 0.7)). Considering the transport variants, differences in weight loss between layer lines can solely be found for Variant II: HNS showed higher loss in weight (−2.38% ± 1.46%) compared with HNB (−1.3% ± 0.71%) (effect: −0.01603; 95% CI: (−0.02026; −0.01180)). Differences in relative weight losses between flocks hardly existed (Figure 6). None of the mean temperature variables on the rearing farm, the transport vehicle, and the farm of laying hens showed a significant effect on the change in body weight.

Birds of the control flock, which were not transported and were kept in the winter garden overnight, free to move around without access to food and water, showed a mean weight loss of −5.9% (95% CI: (−6.3; −5.6)). HNB hens of the control flocks lost significantly less weight (−5.4%; 95% CI: (−5.8; −5.0)) than HNS hens (−6.5%; 95% CI: (−6.9; −6.1)). Comparing the weights of all birds (study and control flocks) calculated as means, birds of the control flock showed on average a higher loss of −2.0% (95% CI: (−2.5; −1.6)) than birds of the study flocks. 

**Figure 7:**
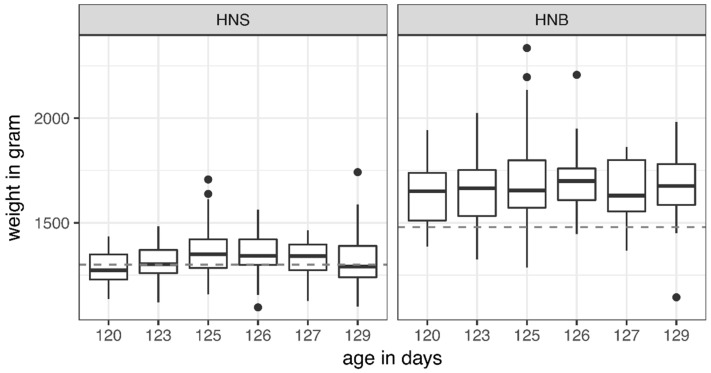
Body weight before transportation in two layer lines, according to age in days. Thick black lines show mean values, boxes represent upper and lower quartiles, whiskers represent 95% confidence intervals, and dots show outliers. The dashed lines represent the target weight according to H&N International for layer lines H&N Super Nick (HNS; 1300 g) and H&N Brown Nick (HNB; 1479 g).

The target weight of the 18-week-old pullets, which is defined by the breeder and distributor H&N International, is set at 1300 g for HNS and 1479 g for HNB. It was not reached by all weighed hens: HNS hens weighed on average 1339 ± 102 g (mean ± SD), and HNB hens 1679 ± 156 g. With age included, no differences could be found within each layer line in reaching the target weight (Figure 7). The only significant effect was the effect of the layer line. The chance of HNS observing a shortfall was on average elevated by a factor of 8.1 (95% CI: (5.1; 12.7)).

## 4. Discussion

To the best of our knowledge, this is the first study comparing two practice-oriented transport variants for pullets over a period of 72 h via fecal CM. The aim of the study was to evaluate which of the two examined transport variants resulted in less pronounced stress responses of the birds. Measurements of CM, a reliable indicator of stress [17], showed no significant transport-specific difference between Variants I and II. Instead, we discovered significant differences between the layer lines in CM responses, touch test results, and weight loss. Several studies have already shown differences between white and brown hens; for example, differences in plasma corticosterone responses after a treatment [33], in tonic immobility [34,35,36,37], or in results from other fear tests [38,39].

### 4.1. Corticosterone Monitoring

Baseline values of CM concentrations and values measured between 0 h and 72 h after transportation did not show significant differences between the two transport variants. Instead, we found significant differences between the two layer lines in both baseline values and values measured after transportation. However, other studies found similar baseline plasma corticosterone concentrations in brown and white layer lines [33,40]. One study analyzed translocation stress in ISA Brown (name of hybride) hens for 36 h after a 1 h long transportation, and found the highest plasma corticosterone concentrations 4 h after transportation [23]. In contrast to this finding, the HNS and HNB hens of our study showed a rapid decrease in CM concentration during the first 6 h after transportation, but just a few returned to baseline values at the end of the study, which might be due to the novel environment. HNS had higher CM levels than HNB at all times in almost every flock. Fraisse and Cockrem [33] reported similar results after 15 min of repeated handling. White hens of their study also showed higher corticosterone levels than brown hens, but only for plasma corticosterone, whereas fecal CM concentrations did not differ between layer lines. At 9 h and 12 h after transportation, CM concentrations in Variant I of our study showed an increase from 61 ng/g at 6 h after transportation, to 96 ng/g and 127 ng/g, respectively, with an intermittent decrease at 10 h (69 ng/g), followed by a steady decline (Table 3). Samples at 9 h and 12 h were taken solely from Flocks N1 and N2; for Flocks N3 to N8, we reduced the sample collection to once at 10 h after transportation. During the investigation period of 72 h, CM concentrations never fell below the value measured 6 h after transportation. In Variant II, we found slight fluctuations of CM concentrations at 34 h and 58 h (additionally taken samples), indicating natural variation due to the circadian rhythm during a 24 h interval. De Jong et al. [41] found a plasma corticosterone peak at 4 h of the 8 h light period during a 24 h investigation on 5-week-old broilers that were fed ad libitum and showed low plasma corticosterone levels during the dark period for 12 h. This finding is contrary to the results from Variant II of our study because samples at 24 h, 48 h and 72 h were taken during the dark period (between 11:00 p.m. and midnight) and samples at 34 h and 58 h were taken during the light period (9–10:00 a.m.). Differences between individual flocks might be attributed to the so-called “passage effect”: Management and processes of transportation, for example, differed. Further investigations are necessary to better understand these differences. 

### 4.2. Hen-Human Relationship: Touch Test

The relationship between animals and humans is an important aspect of animal welfare. Additional contact to humans can positively influence the hen–human relationship [28]. Studies on laying hens showed that additional positive contact with a person resulted in reduced fear toward this person [25,28,42] and in a decrease of plasma corticosterone levels [25]. The pullets of our study behaved contrarily to these findings: Pullets with increased CM concentrations were more likely to allow touch by the test person than pullets with low CM concentrations. Four flocks deviated from the average test results. However, these flocks did not show deviations in any of the other study parts. We therefore cannot relate the tameness of these flocks to other test results of this study.

### 4.3. Body Condition

The examined flocks were in good condition before and after transportation, as measured by the use of the sum of the body parts that were individually scored for plumage condition and integument injuries [43]. With regard to the temporal effect (before and after transportation), we noted a slight improvement to both the plumage and integuments. The main reason is likely to be an insufficient sample size, to ensure that representative estimates and observer deviations are conceivable.

Birds lose weight overnight, even without transportation, and this has potential side effects such as increased corticosterone levels or heat stress, as results from our control flock show. Birds of the control flock were able to move around in the winter garden without access to food and water, matching the lack of these resources for transported birds. “Time in winter garden” for the control flock was 11 h, and this is thus based on the “time in plastic crates” of Variant I. Several studies describe a diurnal and seasonal weight fluctuation in wild birds (e.g., [44,45,46,47]) with amplitudes of 5–15% [44,45,47]. The weight loss of the hens of our control flock (−5.9%) falls within this range. However, a mean loss of −3.9% ± 1.8% overnight, as measured for the transported hens of Variant I, is lower than the loss measured in wild birds. Amplitudes in winter (long and colder nights) are higher than in summer [47]. The mean temperature during transport of the studied flocks was 16 °C, whereas the mean outside temperature for the control flock was 19.5 °C. Unfortunately, none of our other study experiments were performed on the control flock. Explanations therefore remain speculative. Scholtyssek et al. [48] found an greater loss of weight in broilers with increasing durations of transportation (1.3%, 2.3%, and 3.1% after transit durations of 1.5 h, 3.0 h and 4.5 h, respectively) whereas another study did not find weight differences between the control and 4 h transported treatment groups [40].

## 5. Conclusions

Our findings prove that no significant differences exist between the two studied transport variants. This conclusion may be supported by further investigations. Considering the tested flocks, we can say that both transport variants exerted a similar level of stress on the birds. Significant differences between the two layer lines indicated that HNS hens would benefit from transportation in the short variant, whereas stress levels in HNB hens were similar in both variants. Nonetheless, we cannot say whether a longer time of transportation exerts more and longer lasting negative impacts than a shorter period of transportation. Future studies comparing weight development or egg production and egg weight between both transport variants could help to answer the remaining questions.

## Figures and Tables

**Table 1:** Comparison between Switzerland, Germany and Austria regarding maximum stocking densities of pullets and laying hens per pen, as well as minimum space requirements and maximum duration in the transit of pullets according to transnational (European Union, EU), federal, and label-specific regulations. STS = Swiss Animal Protection (“Schweizer Tierschutz”). All numbers in square brackets are references.

Regulation	Country level							
	Switzerland			Germany		Austria		EU
	Federal	Label: Bio Suisse	Label: KAGfreiland	Federal	Label: Demeter, Bioland, Naturland	Federal	Label: Bio Austria	
Maximum Stocking Density Per Pen								
Pullets	No specification [4]	4000 [9]	4000 [10]	No specification [5]	3150 [6]	4800 [18]	4800 [19]	4800 [20]
Layers	18,000 [4]	2000 [9]	2000 [10]	6000 [5]	3000 [6,7,8]	3000 [18]	3000 [19]	3000 [20]
Transport								
Minimum Space in Transport Cage	160 cm^2^/kg for <3.0 kg [4]	According to Transport Guideline of STS [9]	According to Transport Guideline of STS [10]	200 cm^2^/kg for <1.0 kg 190 cm^2^/kg for <1.3 kg 180 cm^2^/kg for <1.6 kg 170 cm^2^/kg for <2.0 kg [5]	No specification [6,7,8]	180–200 cm^2^/kg for <1.6 kg 160 cm^2^/kg for <3.0 kg [18]	180–200 cm^2^/kg for <1.6 kg 160 cm^2^/kg for <3.0 kg [19]	180–200 cm^2^/kg for <1.6 kg 160 cm^2^/kg for <3.0 kg [20]
Maximum Transport Duration	8 h [4]	According to Transport Guideline of STS [9]	No specification for pullets [10]	No specification for pullets [5]	4 h [6,7,8]	No specification for pullets [18]	6 h [19]	12 h [3]

**Table 2:** Comparison of baseline levels (mean ± SEM) of corticosterone metabolites measured in transport Variants I and II in H&N Super Nick (HNS) and H&N Brown Nick (HNB) animals, and in total for both layer lines.

Variant	Line	Baseline Value (ng/g)	SEM (ng/g)	Baseline Value Total (ng/g)	SEM (ng/g)
I	HNS	57	3	43	2
	HNB	30	2		
II	HNS	93	8	66	4
	HNB	40	3		

**Table 3:** Minimum (Min), maximum (Max), and mean concentrations ± SEM of corticosterone metabolites (CM) after transportation in two transport variants.

Variant	Hours after Transportation	Min CM (ng/g)	Max CM (ng/g)	Mean CM (ng/g)	SEM (ng/g)
I	0	4	1337	173	22
	3	4	570	111	7
	6	3	440	61	7
	9	5	568	96	5
	10	4	459	69	6
	12	11	315	128	3
	24	4	786	92	4
	48	3	402	73	3
	72	2	245	64	3
II	0	4	2215	323	11
	3	4	967	134	5
	6	3	992	112	3
	10	4	590	89	11
	24	5	632	95	4
	34	4	307	67	9
	48	5	462	86	5
	58	4	330	69	3
	72	4	456	74	2
